# Association between triglyceride-glucose index and endometriosis: results from a cross-sectional study and Mendelian randomization study

**DOI:** 10.3389/fendo.2024.1388570

**Published:** 2025-01-09

**Authors:** Ting Xu, Yuan Zhuang, Huabin Cao, Jingqi Yang

**Affiliations:** ^1^ Department of Ambulatory Surgical Center, Jiangxi Maternal and Child Health Hospital, Maternal and Child Health Hospital of Nanchang Medical College, Nanchang, Jiangxi, China; ^2^ Department of Cardiovascular Medicine, Jiangxi Provincial People’s Hospital, The First Affiliated Hospital of Nanchang Medical College, Nanchang, Jiangxi, China

**Keywords:** triglyceride-glucose index, endometriosis, Mendelian randomization, cross-sectional study, positive correlation

## Abstract

**Background:**

Triglyceride-glucose (TyG) index has been found to be associated with female reproductive disorders, but its relationship with the risk of endometriosis is unknown. Therefore, the aim of this study was to investigate this relationship.

**Methods:**

We performed a two-sample mendelian randomization(MR) analysis to examine the causal effect of TyG index on endometriosis, and inverse variance weighting(IVW) was the main method of analysis. Then, we selected 1484 participants with endometriosis from the National Health and Nutrition Examination Survey (NHANES) from 1999 to 2006. Weighted multivariable-adjusted logistic regression and smoothed curve analysis were used to evaluate the correlation between the TyG index and endometriosis.

**Results:**

The results of MR analysis confirmed that higher TyG index was causally associated with the risk of endometriosis (OR=1.27, 95%CI: 1.05-1.54, P=0.01). In the cross-sectional study, subjects in the highest quartile of TyG index had the highest risk of incident endometriosis after adjusting for covariates(OR = 2.41, 95% CI:1.31-4.44, P for trend <0.01). The smoothed curve analysis also revealed a positive linear correlation between TyG index and endometriosis.

**Conclusion:**

Our study confirms that a higher TyG index is associated with an increased risk of endometriosis by MR analysis and cross-sectional study. These findings suggested that TyG index could serve as a biomarker in identifying individuals who may be at a higher risk for developing endometriosis. Further research is needed to explore the potential clinical implications of these findings and to elucidate the underlying mechanisms behind this observed relationship.

## Introduction

Endometriosis is a complex oestrogen-dependent chronic inflammatory disease characterized by the appearance of endometrial tissue (glands and mesenchyme) outside the uterus (1). As a common benign gynaecological disorder, it has a prevalence of up to 10% in women of childbearing age and an even higher prevalence in patients with infertility or chronic pelvic pain (2). Endometriosis is strongly associated with pelvic pain, dysmenorrhoea and infertility, imposing a huge economic and social burden on individuals, families and society (3). Despite the numerous studies that have been conducted, endometriosis is considered a multifactorial disease whose etiology is not yet fully understood. Inflammatory responses, oxidative stress, endocrine metabolism and immune regulation have been reported to be involved in the pathological process of endometriosis (4–6).

Research indicates that elevated insulin levels, acting as a growth factor, may stimulate the proliferation of endometrial lesions, and the inflammatory and angiogenic effects of insulin resistance could contribute to the progression of endometriosis (7, 8). This metabolic dysfunction might also explain the variable clinical manifestations in endometriosis patients, underscoring the need for further investigation into the metabolic aspects of endometriosis and the possibility of targeted therapeutic interventions (9). Triglyceride Glucose (TyG) index, as a novel, simple and sensitive index for assessing insulin resistance, calculated from serum triglyceride and blood glucose levels, has been widely used for early screening and risk assessment of metabolic diseases (10, 11). In recent years, many studies have shown a close association between TyG index and various gynaecological diseases, such as polycystic ovary syndrome (12), infertility (13) and gynaecological cancers (14), but there are few systematic and in-depth studies on the relationship between TyG index and endometriosis. Although preliminary studies have suggested that some degree of abnormalities in glycolipid metabolism may exist in patients with endometriosis (15, 16), whether the TyG index can be used as a new marker to predict the risk of endometriosis development and its specific mechanism of action in the pathogenesis of endometriosis needs to be further explored.

Mendelian randomization (MR), which uses genetic variation as an instrumental variable for exposure, has been widely used to explore the link between exposure and outcome and can be a good complement for observational studies (17). Therefore, we first explored the causal relationship between TyG index and endometriosis through MR study. Then, a cross-sectional study was used to explore the correlation between TyG index and endometriosis using the data from the National Health and Nutrition Examination Survey (NHANES) from 1999 to 2006. It will provide a new theoretical basis and clinical strategy for the early prediction and individualized treatment of endometriosis, filling the current research gap in this field.

## Materials and methods

### Data sources and SNP in exposure and outcome selection

The MR analysis was designed based on the following three basic assumptions: (1) the instrumental variable was strongly correlated with the exposure factor; (2) the instrumental variable was not associated with any potential confounders; (3) the instrumental variable was not directly related to the outcome, and its effect on the outcome was manifested only through the exposure. The two-sample MR analysis was used to assess the causal relationship between TyG index and endometriosis.

The genome-wide association study (GWAS) data for TyG index was extracted from UK Biobank database (273368 participants who were aged 40–69 and free from diabetes mellitus and lipid metabolism disorders) (18). The effects of the instrumental SNPs were acquired at the genome-wide level of significance (P < 5 × 10-8) by using linear regression adjusted for age, sex, and the top 5 genetic principal components to control population stratification. After removing SNPs with linkage disequilibrium (R2<0.01) and with glucose or triglyceride, a total of 192 SNPs associated with the TyG index were included in the analysis. Data for endometriosis were obtained from FinnGen database (8288 cases and 68969 controls) (19). To minimize possible bias due to population heterogeneity, all participants come from European.

### MR analysis

The “TwoSampleMR” R package (version 0.5.6, https://github.com/MRCIEU/TwoSampleMR) was used for two-sample MR analysis between TyG and endometriosis. Five MR methods were used: inverse-variance weighted (IVW), MR-Egger regression, and weighted median estimator (WME), simple mode, and weighed mode. We conducted IVW as the primary analysis method. It calculated the Wald ratio for each SNP to evaluate the causality. MR-Egger regression and MR-PRESSO were used to test the pleiotropic effects, and P > 0.05 was regarded as having no pleiotropic effects (20, 21). Heterogeneity was tested using the Cochran’s Q-statistic. If the P-value of Cochran’s Q statistic was >0.05, the results of the random effect IVW method was used, otherwise, the fixed effect model was used. In addition, sensitivity analysis was performed by Leave-one-out method.

### Study population in NHANES

NHANES (https://wwwn.cdc.gov/nchs/nhanes/Default.aspx) is a survey that uses a nationally representative sample to examine the nutritional and health conditions of the civilian population in the United States and is a two-year cross-sectional study conducted by the National Center for Health Statistics (NCHS), Centers for Disease Control and Prevention(CDC). Four consecutive two-year NHANES data (1999-2006) were collected for this study. All data collection methods were approved by the NCHS Research Ethics Review Board and informed consent was obtained from all participants.

**Figure 1:**
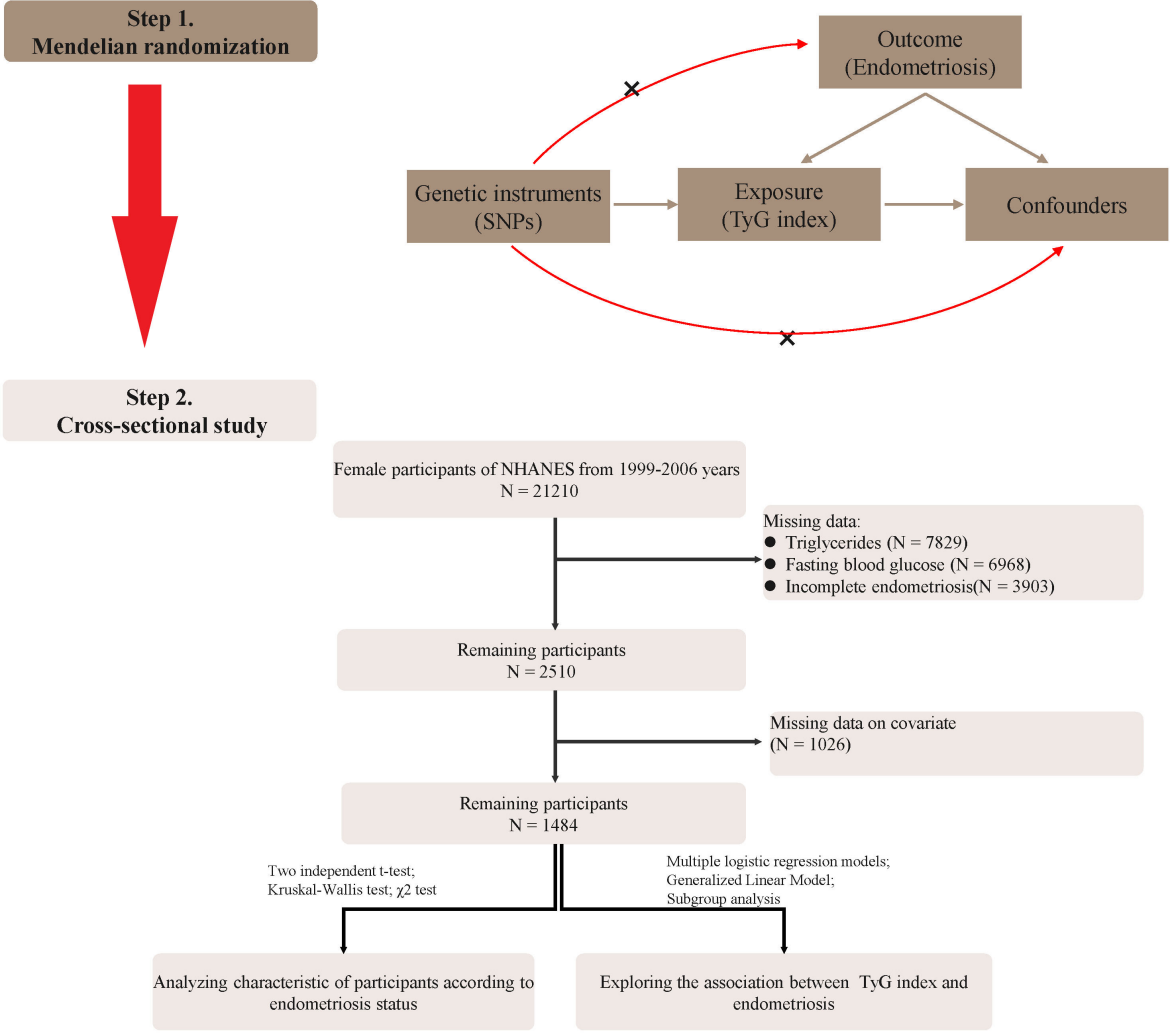
Study flowchart.

We excluded the following individuals: (1) male individuals; (2) individuals lacking data on triglyceride and glucose testing; (3) individuals lacking endometriosis data; (4) pregnant women; and (5) women lacking important covariates. Finally, 1484 participants were enrolled in the study and the flow chart is shown in **Figure 1**.

### Study variables

TyG index was calculated as ln [fasting triglycerides (mg/dL) × fasting glucose (mg/dL)/2] (22). Blood collection was performed in the morning after fasting (at least 8 hours or more but less than 24 hours) to collect fasting triglycerides (TG) and fasting glucose (FPG).

During four cycles of the NHANES 1999 to 2006, female participants were queried about their reproductive health during the Mobile Examination Center (MEC) examination. Participants were asked, ‘Has a doctor or other health professional ever told you that you had endometriosis? Endometriosis is a disease in which the tissue that forms the lining of the uterus/womb attaches to other places, such as the ovaries, fallopian tubes etc.’. Based on these responses, a binary variable indicating the presence or absence of a history of endometriosis diagnosis was established (23).

### Other variables

With reference to recent relevant studies (23, 24), covariates include the following variables: sociodemographic factors of age(years), race (Mexican-American, non-Hispanic white, non-Hispanic black, and other races), education(less than high school college, high school or equivalent and college or above), marital status(married, never married and others) and family poverty income ratio(PIR, ≤1, >1 and ≤3, >3), lifestyle factors of smoking status(Never, Now and Former), alcohol consumption(never, every day or nearly every day, 3 to 4 times a week, 1 to 2 times a week, Less than once a week), female reproductive health factors of age at menarche(<13 and ≥13), number of pregnancies(≤ 3 and > 3) and oral contraceptive(Yes and No).

### Statistical analysis

The data from the NHANES 1999-2006 were extracted, combined, processed and analyzed using the R software(vision 4.3.0, http://www.R-project.org) and Empower software(vision 4.1, http://www.empowerstats.net/analysis/). The weighted t-test or Kruskal-Wallis test was used to analyze the relationship between continuous variables and the weighted chi-square test was used for categorical variables. To analysis the association between TyG index and endometriosis, TyG index was divided into quartiles from lowest (Q1) group to highest (Q4) group. Before modeling, we calculated the variance inflation factor to check for potential multicollinearity (25). Multivariate logistic regression analysis was used to construct three regression models: Model I was no adjustment for any covariates, Model II was adjusted for age, BMI, and race, and Model III was adjusted for all covariates, including age, BMI, Race, Education, PIR, hypertension, diabetes, smoking, drinking, marital status, number of pregnancy, age at Menarche, and oral Contraceptive. Based on the fully adjusted model, we further fitted the relationship between TyG index and the risk of endometriosis by restricted cubic spline (RCS) analysis. Finally, subgroup analysis and interaction test were performed to determine the role of covariates between different TyG index and endometriosis. The P-value < 0.05 was considered statistically significant difference.

## Results

### Causal effects of TyG index on endometriosis

**Figure 2:**
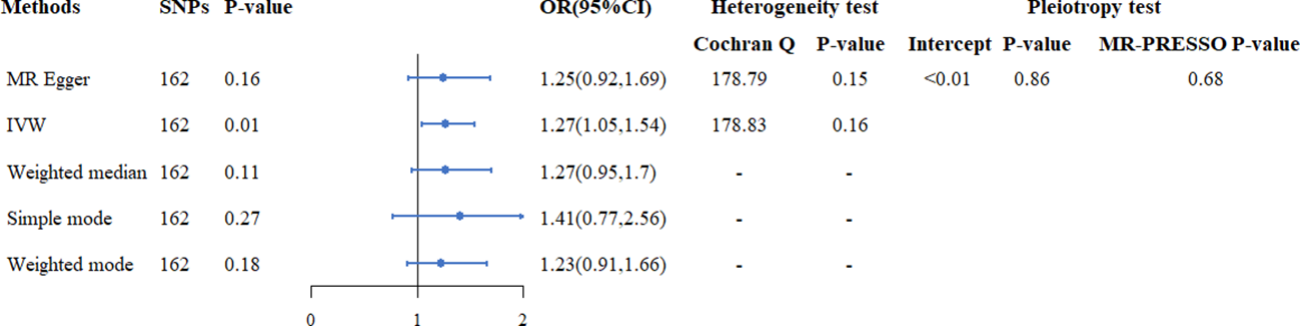
The causal relationship between TyG index and endometriosis in Mendelian randomization study.

The flowchart of our study was shown in **Figure 1**. In the two-sample MR analysis, 162 SNPs were extracted with TyG as the exposure and endometriosis as the outcome. No heterogeneity was found in Cochran’s Q test (P>0.05), therefore, a fixed effects model was used. The result of IVW analysis was found that genetically predicted TyG index was significantly positively associated with endometriosis(OR=1.27, 95%CI=1.05-1.54, P=0.01) (**Figure 2**; **Supplementary Figure S1**). MR-Egger regression (P = 0.86) and MR-Presso global test (P = 0.68) showed no horizontal pleiotropy(**Figure 2**). In addition, the robustness of results was confirmed by the leave-one-out sensitivity test (**Supplementary Figure S2**).

### General characteristics of the study population

The comparison of baseline characteristics between endometriosis and non-endometriosis women was shown in **Table 1**. We found that participants in the endometriosis group were more likely to be had an older age, had a higher percentage of non-Hispanic Whites, had higher levels of education, and had higher percentage of smoking and oral contraceptive use (P < 0.05).

**Table 1:** Baseline characteristics of the participants.

Participant characteristic	Non-endometriosis (N=1353)	Endometriosis (N=131)	P-value
Age	38.86 ± 9.35	41.62 ± 7.89	0.002
BMI	29.28 ± 7.45	29.01 ± 6.80	0.970
Race (%)			<0.001
Mexican American	320 (23.65%)	8 (6.11%)	
Other Hispanic	63 (4.66%)	3 (2.29%)	
Non- Hispanic White	581 (42.94%)	92 (70.23%)	
Non- Hispanic Black	334 (24.69%)	24 (18.32%)	
Others	55 (4.07%)	4 (3.05%)	
Education (%)			0.004
Less than high school	357 (26.39%)	18 (13.74%)	
High school or equivalent	315 (23.28%)	41 (31.30%)	
College or above	681 (50.33%)	72 (54.96%)	
PIR (%)			0.172
<1	261 (19.29%)	21 (16.03%)	
>=1, <3	535 (39.54%)	45 (34.35%)	
>=3	557 (41.17%)	65 (49.62%)	
Marital status (%)			0.489
Married	814 (60.16%)	82 (62.60%)	
Never married	161 (11.90%)	11 (8.40%)	
Others	378 (27.94%)	38 (29.01%)	
Smoking status (%)			0.001
Never	814 (60.16%)	57 (43.51%)	
Now	331 (24.46%)	46 (35.11%)	
Former	208 (15.37%)	28 (21.37%)	
Drinking (%)			0.598
Never	449 (33.19%)	44 (33.59%)	
Every day or nearly every day	295 (21.80%)	21 (16.03%)	
3 to 4 times a week	244 (18.03%)	25 (19.08%)	
1 to 2 times a week	320 (23.65%)	36 (27.48%)	
Less than once a week	45 (3.33%)	5 (3.82%)	
Number of pregnancy (%)			0.893
≤ 3	901 (66.59%)	88 (67.18%)	
> 3	452 (33.41%)	43 (32.82%)	
Age at Menarche (%)			0.136
< 13	672 (49.67%)	74 (56.49%)	
≥ 13	681 (50.33%)	57 (43.51%)	
Oral Contraceptive (%)			0.042
No	276 (20.40%)	17 (12.98%)	
Yes	1077 (79.60%)	114 (87.02%)	
TyG index	6.80 ± 0.63	6.99 ± 0.68	0.002

Dates were presented as median (Interquartile range) or n (%). BMI, Body Mass Index; PIR, Poverty Impact Ratio.

### Correlation between TyG index and endometriosis

**Figure 3:**
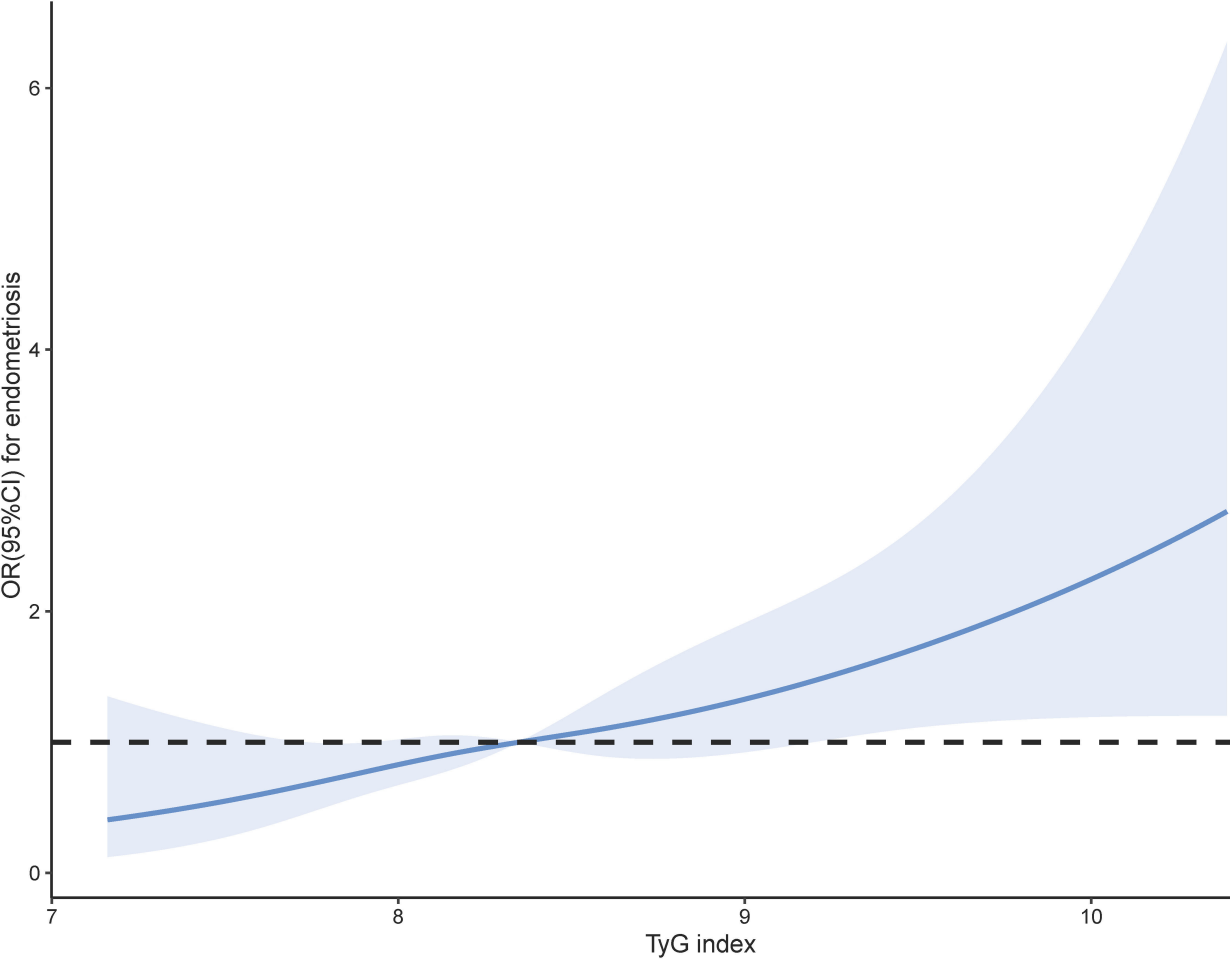
Smoothed curve analysis analysis of TyG index for the estimation of the risk of endometriosis after adjusting for multivariate covariates.

Prior to constructing the multivariable logistic regression analysis, we examined the collinearity between the TyG index and other covariates. The results indicated that the VIF for all covariates included in this study was less than 10, suggesting that there is no collinearity between the TyG index and the other covariates (**Supplementary Table S1**). **Table 2** shows the ORs and 95% CIs of the association between TyG index and endometriosis in the three regression models. In Model I, TyG index showed positive correlations with endometriosis (OR = 1.55, 95% CI: 1.20-2.02). After partial and full adjustment for variables, TyG index still showed a robust positive correlation with endometriosis (OR = 1.60, 95% CI: 1.20-2.12; OR = 1.60, 95% CI: 1.17-2.19, respectively). Based on these results, we further investigated the differences in endometriosis risk between different TyG index quartiles, namely, 5.30-6.36, 6.36-6.75, 6.75-7.19, and 7.20-10.32 in Q1, Q2, Q3, and Q4, respectively. In all three models, it was found that the risk of endometriosis was significantly higher in the Q4 group compared to the Q1 group(Model I: OR = 2.42, 95% CI=1.41-4.13; Model II: OR = 2.39, 95% CI=1.37-4.19; Model III: OR = 2.41, 95% CI=1.31-4.44). In addition, RCS analysis also revealed a positive linear correlation between TyG index and endometriosis (**Figure 3**).

**Table 2:** Association between TyG index and the risks of endometriosis.

	Model I OR(95%CI), P-value	Model II OR(95%CI), P-value	Model III OR(95%CI), P-value
TyG index	1.56 (1.20, 2.02) <0.01	1.60 (1.20, 2.12) <0.01	1.60 (1.17, 2.19) <0.01
TyG index (quartile)			
Q1	1.0	1.0	1.0
Q2	1.57 (0.89, 2.78) 0.12	1.57 (0.88, 2.80) 0.13	1.57 (0.87, 2.82) 0.13
Q3	1.52 (0.86, 2.70) 0.15	1.48 (0.82, 2.66) 0.19	1.49 (0.81, 2.73) 0.20
Q4	2.42 (1.41, 4.13) <0.01	2.39 (1.37, 4.19) <0.01	2.41 (1.31, 4.44) <0.01
P for trend	<0.01	<0.01	<0.01

95% CI, 95% confidence interval; OR, odds ratio.

Model I adjust for: None.

Model II adjust for: Age; BMI; Race.

Model III adjust for: Age; BMI; Race; Education; Poverty-to-income ratio; Marital status; Drinking; Smoking; Number of pregnancy; Age at Menarche; Oral Contraceptive.

### Subgroup analysis

**Figure 4:**
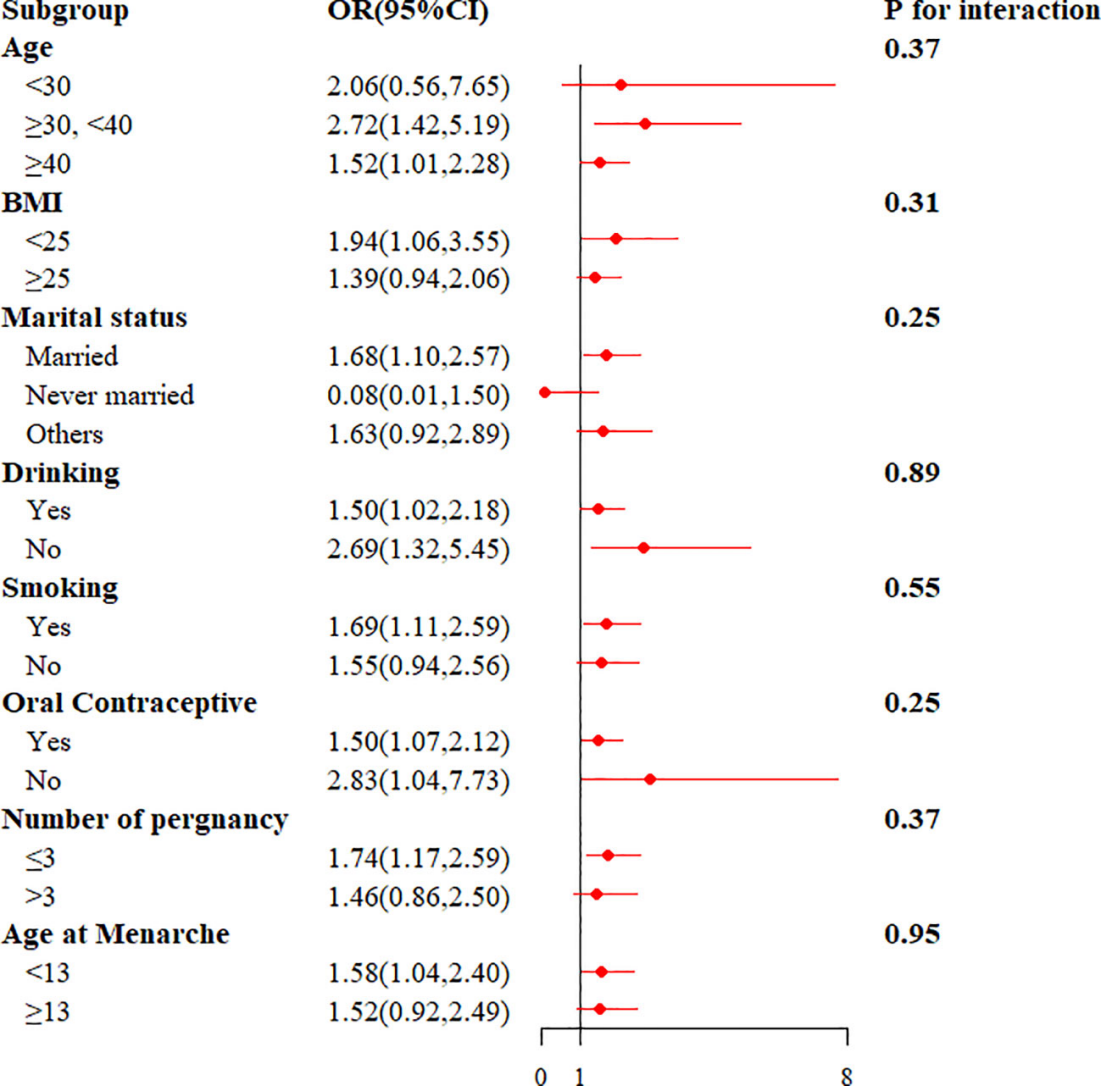
The results of stratified analysis for the association between TyG index and endometriosis.

To study the relationship between the TyG index and endometriosis in different populations, we conducted subgroup analyses. The TyG index was positively associated with the risk of endometriosis in those aged ≥30 years, BMI <25, married, smoking, number of pregnancies ≤3, and age at menarche <13 (P < 0.05). In addition, interaction tests did not show statistically significant variations in the association between TyG index and endometriosis, indicating that the covariates were no significantly dependent on this positive correlation (all interactions P > 0.05) (**Figure 4**).

## Discussion

The present study aimed to investigate the relationship between TyG index and the risk of endometriosis by combining the cross-sectional study of NHANES 1999-2006, and the two-sample MR analysis. The cross-sectional study revealed a positive correlation between TyG index and the risk of endometriosis, and more importantly, MR analysis have shown a causal relationship between. These findings may provide additional information to predict the occurrence of endometriosis in the female population.

There are many studies on the relationship between TyG index and female reproductive diseases (12–14), but studies on TyG index and endometriosis and the causal relationship between them have not been elucidated. To the best of our knowledge, our study is the first to comprehensively assess the relationship between TyG index and endometriosis. In the MR analysis, we used GWAS data of TyG index sourced from UK Biobank as an exposure and extracted 192 SNPs to be analyzed for causality. In our analyses, no heterogeneity was found by the Cochran Q test, as well as no horizontal pleiotropy of effects by MR-Egger regression and MR-Presso global test. Thus, the application of a fixed-effects model for IVW suggested that the genetic risk of TyG index was directly associated with the risk of endometriosis. Sensitivity tests also support the stability and accuracy of the causal results.

The observed positive association between TyG index and endometriosis in our study raises several important considerations. The development of endometriosis is multifactorial, involving genetic, hormonal, and environmental factors. Recent research has highlighted the potential role of metabolic factors in the pathogenesis of endometriosis (9). The TyG index, which combines triglycerides and FPG, reflects insulin resistance and is considered a useful marker for metabolic disorders such as diabetes (26) and cardiovascular diseases (27). In fact, endometriosis has been proposed as a metabolic disorder by Saunders et al (28). In a prospective cohort study, metabolomic analysis using proton-nuclear magnetic resonance revealed higher concentrations of free fatty acids and lipids in the serum metabolic profiles of patients with endometriosis (29). Another retrospective cohort study demonstrated that disorders of glucose metabolism have an impact on the development and outcome of endometriosis (30). It suggested that metabolic dysfunction may play a role in the pathogenesis of endometriosis. Non-hormonal therapy based on metabolic changes may be a novel therapeutic option for the treatment of endometriosis (31). Additionally, insulin resistance is often accompanied by hyperinsulinemia, which leads to increased levels of free insulin-like growth factor-1 (IGF-1), a known promoter of endometrial cell growth (7, 32, 33). These factors may create a conducive environment for the establishment and growth of ectopic endometrial tissue.

One potential mechanism underlying the association between TyG index and endometriosis is chronic inflammation. Insulin resistance, as indicated by the elevated TyG index, can trigger a pro-inflammatory state (34), which may contribute to the development and progression of endometriosis (35). Previous studies have shown that inflammation is closely related to the establishment and maintenance of endometriotic lesions (36), and that elevated levels of pro-inflammatory cytokines are present in the peritoneal fluid of women with endometriosis (37, 38). This inflammation can lead to a cascade of events that not only exacerbate insulin resistance but also serve to exacerbate the development and progression of endometriosis. Moreover, insulin resistance itself may have direct effects on endometrial tissue (39). Insulin, as a growth factor, can stimulate the proliferation of endometrial cells, potentially promoting the implantation and growth of ectopic endometrial tissue (40, 41). Insulin resistance may also disrupt the balance of hormones involved in endometrial homeostasis, further contributing to the development of endometriosis (42, 43). Future research should focus on elucidating the specific molecular mechanisms by which chronic inflammation and insulin resistance interact to influence endometrial cell behavior.

The study included the MR analysis and the cross-sectional study based on NHANES 1999-2006 whose findings corroborate each other to a high degree of confidence. This approach compensates for the shortcomings of observational study and minimizes potential confounding effects. Despite the significant findings of our study, there are several limitations that need to be addressed. Firstly, the diagnosis of endometriosis was collected through questionnaires based on previous professional medical diagnoses, which may underestimate the number of affected individuals as some people might not have sought professional medical evaluation. Second, the reliance on questionnaires could introduce memory and selection biases, potentially impacting the results. Third, the cross-sectional data on endometriosis were collected between 1999 and 2006, and although the NHANES database updates every two years, this particular questionnaire was not included after 2006, which may affect the timeliness and thus the outcomes of our study. Fourth, it is challenging to rule out selection bias in this study, as a significant portion of the population was excluded from the cross-sectional analysis due to missing variables. Lastly, MR analyses was performed in a European population, which may limit the extrapolation of our results to other populations. Future studies should include more diverse populations to validate the observed associations.

In conclusion, our findings suggest a positive association between TyG index and endometriosis for the first time. The underlying mechanisms may involve insulin resistance, chronic inflammation, and dysregulation of endometrial homeostasis. Further research is needed to elucidate the potential therapeutic implications of targeting TyG index in the prevention and management of endometriosis. Understanding the relationship metabolic dysfunction and endometriosis could aid in the identification of individuals at risk and facilitate the development of personalized approaches for prevention and treatment strategies.

## Data Availability

Publicly available datasets were analyzed in this study. The data of NHANES can be downloaded from the website: https://www.cdc.gov/nchs/nhanes/index.htm.
