# Supplementary material for: Association between triglyceride-glucose index and endometriosis: results from a cross-sectional study and Mendelian randomization study
Source: Front Endocrinol (Lausanne). 2025 Jan 9;15:1388570. doi: 10.3389/fendo.2024.1388570 (PMC11753958; doi:10.3389/fendo.2024.1388570)
Supplement: Supplementary file 1 [file DataSheet1.docx]

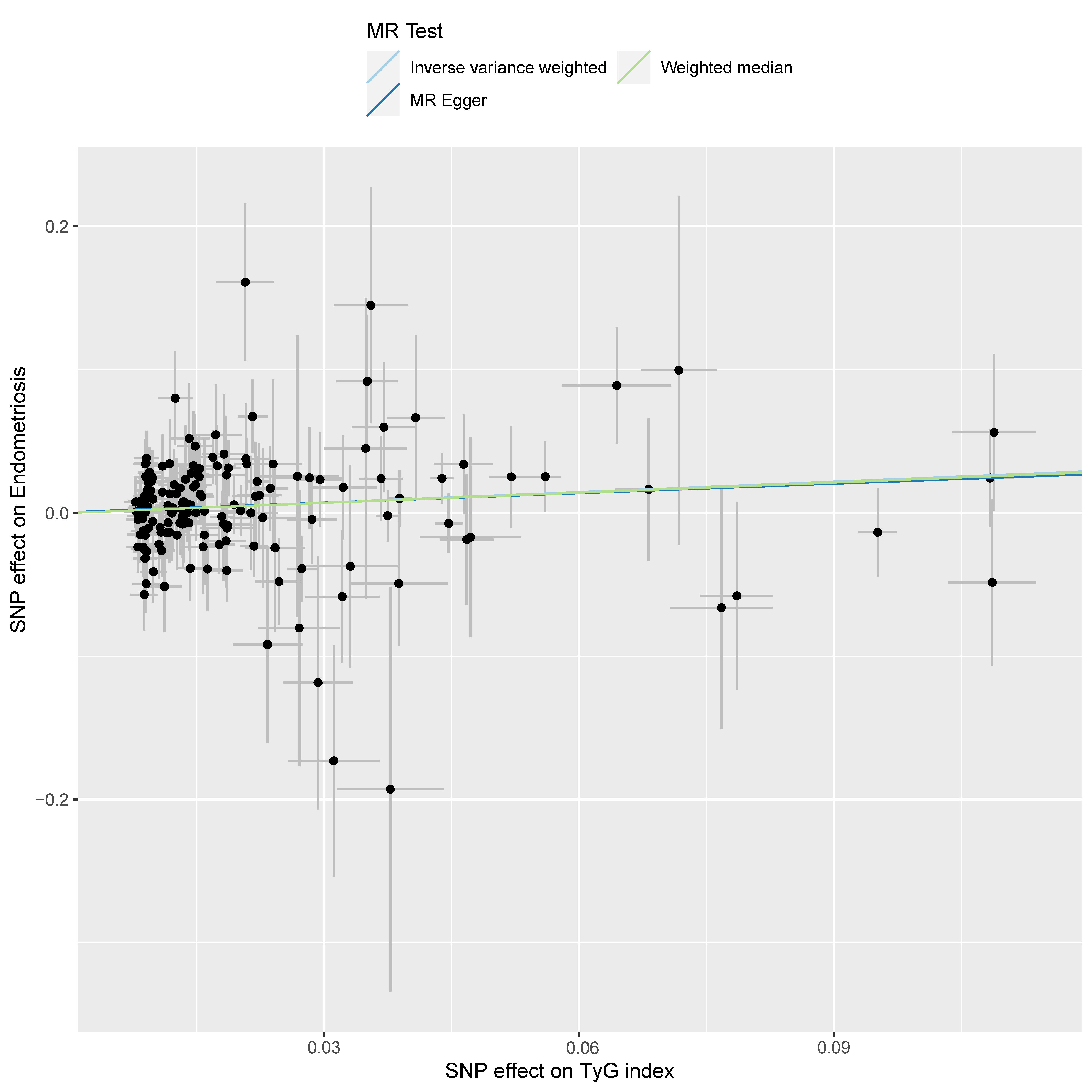


Supplement Figure 1. Scatter plot of genetic correlations of TyG index and endometriosis using different MR methods.


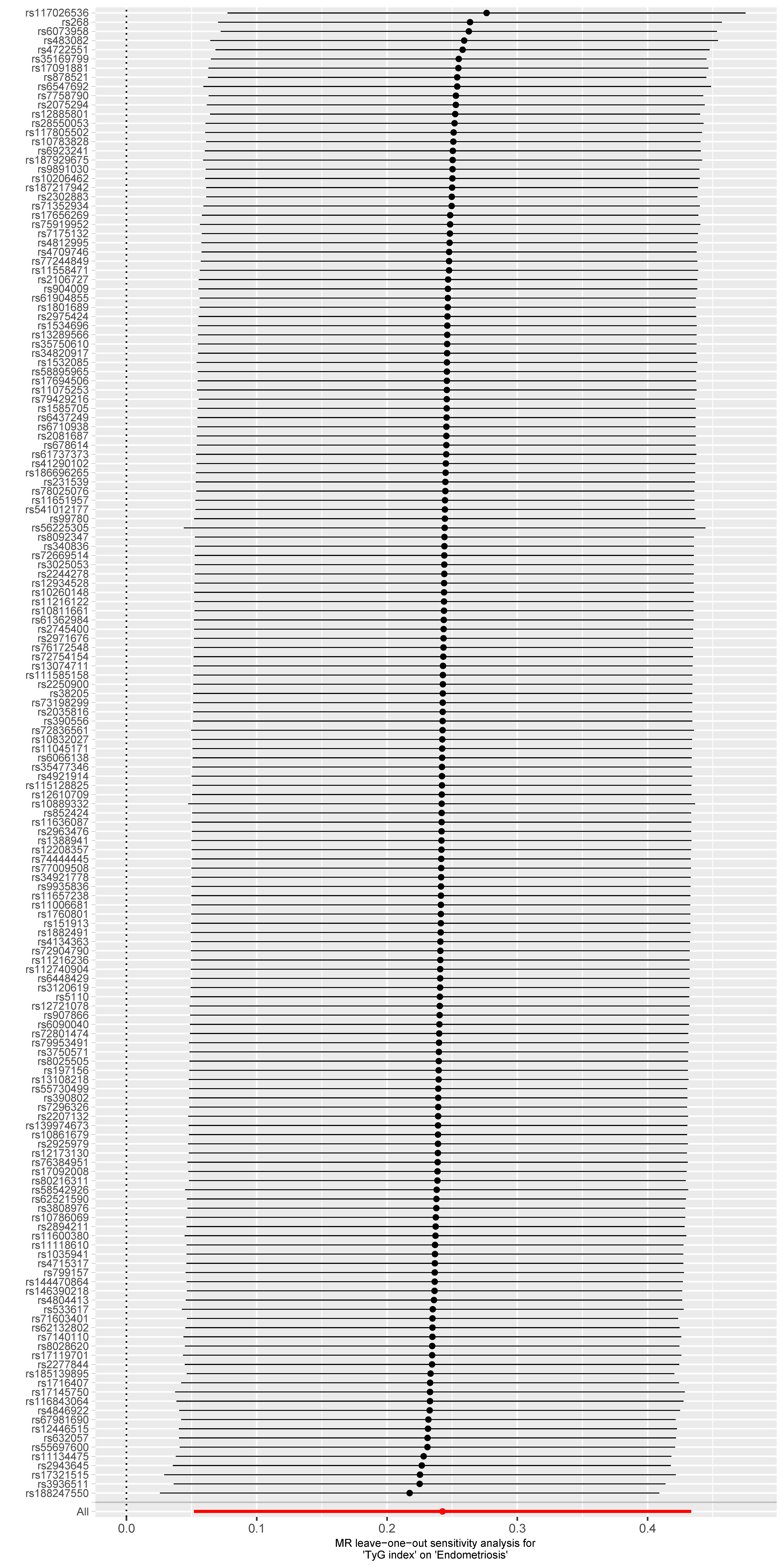


Supplement Figure 2. Forest plot of the causal effects of TyG index associated SNPs on endometriosis.

Supplementary Table 1: Collinearity diagnostics steps.

|  | VIF |  |
| --- | --- | --- |
| TyG | 1.2 | |
| Race | 1.2 | |
| Oral Contraceptive | 1.1 | |
| Education | 1.3 | |
| Number of pregnancy | 1.1 | |
| Marital status | 1.1 | |
| Poverty-to-income ratio | 1.4 | |
| Age at Menarche | 1 | |
| Drinking | 1.1 | |
| Smoking | 1.1 | |
| BMI | 1.2 | |
| Age | 1.2 | |

VIF: variance inflation factor; VIF = 1/(1-R^2^)

Abbreviations are as follows. TyG: Triglyceride-glucose; BMI: Body Mass Index

Note: The variables with VIF>10 will be regarded as collinear variables and cannot be included in the multiple regression model
