# Supplementary material for: Association between triglyceride-glucose index and endometriosis: results from a cross-sectional study and Mendelian randomization study
Source: Front Endocrinol (Lausanne). 2025 Jan 9;15:1388570. doi: 10.3389/fendo.2024.1388570 (PMC11753958; doi:10.3389/fendo.2024.1388570)
Supplement: Supplementary file 2 [file DataSheet2.docx]

**STROBE-MR checklist of recommended items to address in reports of Mendelian randomization studies**^1^ ^2^

| **Item No.** | **Section** | **Checklist item** | **Page No.** | **Relevant text from manuscript** |
| --- | --- | --- | --- | --- |
| 1 | **TITLE and ABSTRACT** | Indicate Mendelian randomization (MR) as the study’s design in the title and/or the abstract if that is a main purpose of the study | 1 | Association between triglyceride-glucose index and endometriosis: Results from a cross-sectional study and Mendelian Randomization study |
|  | **INTRODUCTION** |  |  |  |
| 2 | **Background** | Explain the scientific background and rationale for the reported study. What is the exposure? Is a potential causal relationship between exposure and outcome plausible? Justify why MR is a helpful method to address the study question | 2 | Mendelian randomization (MR), which uses genetic variation as an instrumental variable for exposure, has been widely used to explore the link between exposure and outcome and can be a good complement for observational studies |
| 3 | **Objectives** | State specific objectives clearly, including pre-specified causal hypotheses (if any). State that MR is a method that, under specific assumptions, intends to estimate causal effects | 2 | we first explored the causal relationship between TyG index and endometriosis through MR study |
|  | **METHODS** |  |  |  |
| 4 | **Study design and data sources** | Present key elements of the study design early in the article. Consider including a table listing sources of data for all phases of the study. For each data source contributing to the analysis, describe the following: |  |  |
|  | a) | Setting: Describe the study design and the underlying population, if possible. Describe the setting, locations, and relevant dates, including periods of recruitment, exposure, follow-up, and data collection, when available. | 3 | The genome-wide association study (GWAS) data for TyG index was extracted from UK Biobank database (273368 participants who were aged 40–69 and free from diabetes mellitus and lipid metabolism disorders). The effects of the instrumental SNPs were acquired at the genome-wide level of significance (P < 5 × 10-8) by using linear regression adjusted for age, sex, and the top 5 genetic principal components to control population stratification. After removing SNPs with linkage disequilibrium (R2<0.01) and with glucose or triglyceride, a total of 192 SNPs associated with the TyG index were included in the analysis. Data for endometriosis were obtained from FinnGen database (8288 cases and 68969 controls). |
|  | b) | Participants: Give the eligibility criteria, and the sources and methods of selection of participants. Report the sample size, and whether any power or sample size calculations were carried out prior to the main analysis | 3 | The MR analysis was designed based on the following three basic assumptions: (1) the instrumental variable was strongly correlated with the exposure factor; (2) the instrumental variable was not associated with any potential confounders; (3) the instrumental variable was not directly related to the outcome, and its effect on the outcome was manifested only through the exposure. The two-sample MR analysis was used to assess the causal relationship between TyG index and endometriosis |
|  | c) | Describe measurement, quality control and selection of genetic variants | 3 | The MR analysis was designed based on the following three basic assumptions: (1) the instrumental variable was strongly correlated with the exposure factor; (2) the instrumental variable was not associated with any potential confounders; (3) the instrumental variable was not directly related to the outcome, and its effect on the outcome was manifested only through the exposure. The two-sample MR analysis was used to assess the causal relationship between TyG index and endometriosis.  After removing SNPs with linkage disequilibrium (R2<0.01) and with glucose or triglyceride, a total of 192 SNPs associated with the TyG index were included in the analysis. |
|  | d) | For each exposure, outcome, and other relevant variables, describe methods of assessment and diagnostic criteria for diseases | 3 | The genome-wide association study (GWAS) data for TyG index was extracted from UK Biobank database (273368 participants who were aged 40–69 and free from diabetes mellitus and lipid metabolism disorders)(16). The effects of the instrumental SNPs were acquired at the genome-wide level of significance (P < 5 × 10-8) by using linear regression adjusted for age, sex, and the top 5 genetic principal components to control population stratification. |
|  | e) | Provide details of ethics committee approval and participant informed consent, if relevant | 6 | The data of NHANES is public database. The patients involved in the database received ethical approval. Users can download relevant data for free for research and publication purposes. |
| 5 | **Assumptions** | Explicitly state the three core IV assumptions for the main analysis (relevance, independence and exclusion restriction) as well assumptions for any additional or sensitivity analysis | 3 | The MR analysis was designed based on the following three basic assumptions: (1) the instrumental variable was strongly correlated with the exposure factor; (2) the instrumental variable was not associated with any potential confounders; (3) the instrumental variable was not directly related to the outcome, and its effect on the outcome was manifested only through the exposure. |
| 6 | **Statistical methods: main analysis** | Describe statistical methods and statistics used |  |  |
|  | a) | Describe how quantitative variables were handled in the analyses (i.e., scale, units, model) | 3 | The effects of the instrumental SNPs were acquired at the genome-wide level of significance (P < 5 × 10-8) by using linear regression adjusted for age, sex, and the top 5 genetic principal components to control population stratification. |
|  | b) | Describe how genetic variants were handled in the analyses and, if applicable, how their weights were selected | 3 | After removing SNPs with linkage disequilibrium (R2<0.01) and with glucose or triglyceride, a total of 192 SNPs associated with the TyG index were included in the analysis. |
|  | c) | Describe the MR estimator (e.g. two-stage least squares, Wald ratio) and related statistics. Detail the included covariates and, in case of two-sample MR, whether the same covariate set was used for adjustment in the two samples | 3 | The “TwoSampleMR” R package (version 0.5.6, https://github.com/MRCIEU/TwoSampleMR) was used for two-sample MR analysis between TyG and endometriosis. Five MR methods were used: inverse-variance weighted (IVW), MR-Egger regression, and weighted median estimator (WME), simple mode, and weighed mode. We conducted IVW as the primary analysis method. It calculated the Wald ratio for each SNP to evaluate the causality. MR-Egger regression and MR-PRESSO were used to test the pleiotropic effects, and P ˃ 0.05 was regarded as having no pleiotropic effects |
|  | d) | Explain how missing data were addressed | 4 | In the two-sample MR analysis, 162 SNPs were extracted with TyG as the exposure and endometriosis as the outcome. |
|  | e) | If applicable, indicate how multiple testing was addressed |  | Not applicable |
| 7 | **Assessment of assumptions** | Describe any methods or prior knowledge used to assess the assumptions or justify their validity | 3 | MR-Egger regression and MR-PRESSO were used to test the pleiotropic effects, and P ˃ 0.05 was regarded as having no pleiotropic effects(18, 19). Heterogeneity was tested using the Cochran's Q-statistic. If the P-value of Cochran's Q statistic was >0.05, the results of the random effect IVW method was used, otherwise, the fixed effect model was used. |
| 8 | **Sensitivity analyses and additional analyses** | Describe any sensitivity analyses or additional analyses performed (e.g. comparison of effect estimates from different approaches, independent replication, bias analytic techniques, validation of instruments, simulations) | 3 | In addition, sensitivity analysis was performed by Leave-one-out method |
| 9 | **Software and pre-registration** |  |  |  |
|  | a) | Name statistical software and package(s), including version and settings used | 3 | The “TwoSampleMR” R package (version 0.5.6, https://github.com/MRCIEU/TwoSampleMR) was used for two-sample MR analysis between TyG and endometriosis |
|  | b) | State whether the study protocol and details were pre-registered (as well as when and where) |  | The study protocol and details were not pre-registered |
|  | **RESULTS** |  |  |  |
| 10 | **Descriptive data** |  |  |  |
|  | a) | Report the numbers of individuals at each stage of included studies and reasons for exclusion. Consider use of a flow diagram | 4 | The flowchart of our study was shown in Figure 1 |
|  | b) | Report summary statistics for phenotypic exposure(s), outcome(s), and other relevant variables (e.g. means, SDs, proportions) | 3 | The genome-wide association study (GWAS) data for TyG index was extracted from UK Biobank database (273368 participants who were aged 40–69 and free from diabetes mellitus and lipid metabolism disorders)(16). The effects of the instrumental SNPs were acquired at the genome-wide level of significance (P < 5 × 10-8) by using linear regression adjusted for age, sex, and the top 5 genetic principal components to control population stratification. After removing SNPs with linkage disequilibrium (R2<0.01) and with glucose or triglyceride, a total of 192 SNPs associated with the TyG index were included in the analysis. Data for endometriosis were obtained from FinnGen database (8288 cases and 68969 controls) |
|  | c) | If the data sources include meta-analyses of previous studies, provide the assessments of heterogeneity across these studies |  | Data sources do not include meta-analyses of previous studies |
|  | d) | For two-sample MR:  i.  Provide justification of the similarity of the genetic variant-exposure associations between the exposure and outcome samples  ii.  Provide information on the number of individuals who overlap between the exposure and outcome studies | 3 | To minimize possible bias due to population heterogeneity, all participants come from European. |
| 11 | **Main results** |  |  |  |
|  | a) | Report the associations between genetic variant and exposure, and between genetic variant and outcome, preferably on an interpretable scale | 4 | In the two-sample MR analysis, 162 SNPs were extracted with TyG as the exposure and endometriosis as the outcome. |
|  | b) | Report MR estimates of the relationship between exposure and outcome, and the measures of uncertainty from the MR analysis, on an interpretable scale, such as odds ratio or relative risk per SD difference | 4 | The result of IVW analysis was found that genetically predicted TyG index was significantly positively associated with endometriosis(OR=1.27, 95%CI=1.05-1.54, P=0.01)(Figure 2, Supplement Figure1). |
|  | c) | If relevant, consider translating estimates of relative risk into absolute risk for a meaningful time period |  | The study design did not include a time variable, so this transformation could not be performed. |
|  | d) | Consider plots to visualize results (e.g. forest plot, scatterplot of associations between genetic variants and outcome versus between genetic variants and exposure) | 4 | The result of IVW analysis was found that genetically predicted TyG index was significantly positively associated with endometriosis(OR=1.27, 95%CI=1.05-1.54, P=0.01)(Figure 2, Supplement Figure1) |
| 12 | **Assessment of assumptions** |  |  |  |
|  | a) | Report the assessment of the validity of the assumptions | 4 | MR-Egger regression (P = 0.86) and MR-Presso global test (P = 0.68) showed no horizontal pleiotropy(Figure 2). In addition, the robustness of results was confirmed by the leave-one-out sensitivity test (Supplement Figure2). |
|  | b) | Report any additional statistics (e.g., assessments of heterogeneity across genetic variants, such as *I^2^*, Q statistic or E-value) | 4 | In the two-sample MR analysis, 162 SNPs were extracted with TyG as the exposure and endometriosis as the outcome. No heterogeneity was found in Cochran's Q test (P>0.05), therefore, a fixed effects model was used. |
| 13 | **Sensitivity analyses and additional analyses** |  |  |  |
|  | a) | Report any sensitivity analyses to assess the robustness of the main results to violations of the assumptions | 4 | MR-Egger regression (P = 0.86) and MR-Presso global test (P = 0.68) showed no horizontal pleiotropy(Figure 2). |
|  | b) | Report results from other sensitivity analyses or additional analyses | 4 | In addition, the robustness of results was confirmed by the leave-one-out sensitivity test (Supplement Figure2). |
|  | c) | Report any assessment of direction of causal relationship (e.g., bidirectional MR) | 5 | Correlation of TyG index with endometriosis was studied in clinical data |
|  | d) | When relevant, report and compare with estimates from non-MR analyses | 5 | We have analysed and validated the relationship between TyG index and endometriosis in another cross-sectional study. |
|  | e) | Consider additional plots to visualize results (e.g., leave-one-out analyses) | 4 | the robustness of results was confirmed by the leave-one-out sensitivity test (Supplement Figure2). |
|  | **DISCUSSION** |  |  |  |
| 14 | **Key results** | Summarize key results with reference to study objectives | 5 | MR analysis have shown a causal relationship between. |
| 15 | **Limitations** | Discuss limitations of the study, taking into account the validity of the IV assumptions, other sources of potential bias, and imprecision. Discuss both direction and magnitude of any potential bias and any efforts to address them | 6 | Second, MR analyses was performed in a European population, which may limit the extrapolation of our results to other populations. Future studies should include more diverse populations to validate the observed associations |
| 16 | **Interpretation** |  |  |  |
|  | a) | Meaning: Give a cautious overall interpretation of results in the context of their limitations and in comparison with other studies | 6 | There are many studies on the relationship between TyG index and female reproductive diseases(10-12), but studies on TyG index and endometriosis and the causal relationship between them have not been elucidated. |
|  | b) | Mechanism: Discuss underlying biological mechanisms that could drive a potential causal relationship between the investigated exposure and the outcome, and whether the gene-environment equivalence assumption is reasonable. Use causal language carefully, clarifying that IV estimates may provide causal effects only under certain assumptions | 6 | One potential mechanism underlying the association between TyG index and endometriosis is chronic inflammation. |
|  | c) | Clinical relevance: Discuss whether the results have clinical or public policy relevance, and to what extent they inform effect sizes of possible interventions | 6 | The study included the MR analysis and the cross-sectional study based on NHANES 1999-2006 whose findings corroborate each other to a high degree of confidence. |
| 17 | **Generalizability** | Discuss the generalizability of the study results (a) to other populations, (b) across other exposure periods/timings, and (c) across other levels of exposure | 6 | MR analyses was performed in a European population, which may limit the extrapolation of our results to other populations. Future studies should include more diverse populations to validate the observed associations |
|  | **OTHER INFORMATION** |  |  |  |
| 18 | **Funding** | Describe sources of funding and the role of funders in the present study and, if applicable, sources of funding for the databases and original study or studies on which the present study is based | 7 | The authors’ research was supported by Health and Family Planning Commission of Jiangxi Province (Grant Number: 202211101). |
| 19 | **Data and data sharing** | Provide the data used to perform all analyses or report where and how the data can be accessed, and reference these sources in the article. Provide the statistical code needed to reproduce the results in the article, or report whether the code is publicly accessible and if so, where | 8 | 16 Si S, Li J, Li Y, Li W, Chen X, Yuan T, et al. Causal Effect of the Triglyceride-Glucose Index and the Joint Exposure of Higher Glucose and Triglyceride with Extensive Cardio-Cerebrovascular Metabolic Outcomes in the Uk Biobank: A Mendelian Randomization Study. *Front Cardiovasc Med* (2020) 7:583473. Epub 2021/02/09. doi: 10.3389/fcvm.2020.583473.  17. Kurki MI, Karjalainen J, Palta P, Sipilä TP, Kristiansson K, Donner KM, et al. Finngen Provides Genetic Insights from a Well-Phenotyped Isolated Population. *Nature* (2023) 613(7944):508-18. Epub 2023/01/19. doi: 10.1038/s41586-022-05473-8. |
| 20 | **Conflicts of Interest** | All authors should declare all potential conflicts of interest | 6 | The authors have no conflicts of interest to declare. |

This checklist is copyrighted by the Equator Network under the Creative Commons Attribution 3.0 Unported (CC BY 3.0) license.

1. Skrivankova VW, Richmond RC, Woolf BAR, Yarmolinsky J, Davies NM, Swanson SA, et al. Strengthening the Reporting of Observational Studies in Epidemiology using Mendelian Randomization (STROBE-MR) Statement. JAMA. 2021;under review.

2. Skrivankova VW, Richmond RC, Woolf BAR, Davies NM, Swanson SA, VanderWeele TJ, et al. Strengthening the Reporting of Observational Studies in Epidemiology using Mendelian Randomisation (STROBE-MR): Explanation and Elaboration. BMJ. 2021;375:n2233.
